# Clinical Decision-Making in Dental Anaesthesia: Integrating Patient Factors, Procedural Complexity and Outcome Measures

**DOI:** 10.7759/cureus.108818

**Published:** 2026-05-13

**Authors:** Nishant Visvas Dumont, Omkar Eswara Babu Danda, Uttara Patnaik, Pravesh Saraswat, Vedangi Shashikant Patil, Priyanka Tripathi

**Affiliations:** 1 Department of Oral and Maxillofacial Surgery, Pondicherry University, Pondicherry, IND; 2 Department of Conservative Dentistry and Endodontics, Dr. Nandamuri Taraka Rama Rao (NTR) University of Health Sciences, Vijayawada, IND; 3 Department of Hospital Management, Lovely Professional University, Phagwara, IND; 4 Department of Nursing, Medanta Hospital, Noida, IND; 5 Department of Paediatric and Preventive Dentistry, Clove Dental, Navi Mumbai, IND; 6 Department of Dentistry, All India Institute of Medical Sciences, Gorakhpur, Gorakhpur, IND

**Keywords:** anaesthesia, decision-making, dentistry, pain management, sedation

## Abstract

Dental anaesthesia plays a critical role in controlling pain and ensuring patient comfort during dental procedures. The effective administration of anaesthetic agents enables clinicians to perform diagnostic, restorative and surgical treatments while minimising discomfort and anxiety. Advances in pharmacology, anaesthetic techniques and clinical safety protocols have improved the effectiveness and reliability of dental anaesthesia in contemporary practice. This review examines clinical decision-making in dental anaesthesia by integrating patient-related factors, procedural complexity and outcome measures that influence the selection and effectiveness of anaesthetic techniques in dentistry. This comprehensive review studies published literature related to dental pain mechanisms, pharmacological properties of anaesthetic agents, patient assessment and procedural considerations in dental treatment. Studies addressing anaesthetic techniques, monitoring practices and safety protocols in dentistry were examined to provide an integrated overview of clinical decision-making in dental anaesthesia. The literature considered in this review primarily includes studies published between 2015 and 2025, reflecting recent developments and current clinical practices in dental anaesthesia. Dental anaesthetic management is influenced by several factors, including patient health status, psychological conditions, treatment complexity and pharmacological characteristics of anaesthetic agents. Local anaesthetic drugs such as lidocaine, articaine, mepivacaine and bupivacaine remain widely used due to their effectiveness in blocking neural transmission and controlling procedural pain. Careful patient assessment, the continuous monitoring of physiological parameters and adherence to established clinical protocols contribute to improved safety during dental procedures. The consideration of patient characteristics, procedural requirements and pharmacological factors supports effective anaesthetic selection and enhances clinical outcomes in dental practice.

## Introduction and background

Among the most frequent causes of patients seeking dental treatment is dental pain, and it is also a primary health concern across the entire world in terms of quality of life and daily functioning [1]. Oral tissues could be a source of painful experiences that interfere with the process of eating, talking and overall well-being, and so, the effective pain management of the oral tissues is an absolute necessity in the fields of dentistry [2]. Dental practices such as dental restorations, dental extractions and endodontic practices tend to cause tissue manipulation that may lead to discomfort unless adequate control of anaesthesia has been taken [3]. Local anaesthesia is therefore playing a key role in modern-day dentistry by enabling practitioners to carry out their duties safely, besides ensuring that the patient experiences less pain and is less anxious [4]. Efficient pain management also enhances patient cooperation and is linked with good treatment outcomes with routine and complex dental operations [5]. Also, there is another aspect of poor pain management that may discourage patients from receiving timely treatment of dental problems, which also may result in the worsening of oral health conditions and the necessity to use more severe treatment methods [6].

The improvement in dental anaesthetic techniques, as well as pharmacology, has played an important role in the enhanced management of procedural pain over the last few decades [7]. Contemporary dentistry uses anaesthetic forms, such as local anaesthesia, conscious sedation and general anaesthesia, based on the needs of the patient and the nature of the treatment [8]. The most commonly used one is local anaesthesia that leads to a loss of sensation sustained locally and reversibly without the disturbance of the patient's consciousness [9]. Where the patient is too anxious or the surgery is of a major nature, the patient may need to be sedated or put under general anaesthesia so that the treatment can be administered safely and effectively [10]. The safety and reliability of the said procedures have also increased due to improvements in technology in terms of both the system of anaesthetic delivery and the monitoring system of the clinical procedure [11]. This has prompted the thinking that a mix of the treatment of the various forms of anaesthesia employed in contemporary dental practice is necessary to afford the patient comfort, as well as procedural success [12].

To choose the right method of anaesthetic procedure, a proper clinical decision-making must be conducted, considering both patient- and procedure-related issues [13]. One of the duties of dentists is to determine the medical history of their patients, their systemic conditions and potential drug interactions before the administration of an anaesthetic agent [14]. The type of anaesthesia to be applied in dental treatment is also influenced by procedural variables, including the length of the treatment procedure, the complexity of both the surgical procedure and anatomy [15]. Formulated clinical decisions to assist in the selection of safe and effective anaesthetic techniques in specific patients have been progressively suggested [16]. These models assist clinicians in balancing dental procedures and effective pain management with patient safety issues. The evidence-based practices are also supporting the consistency in clinical practice and the reduction of the risk of complications in the administration of anaesthetics.

Medicines that are involved in dental anaesthesia primarily inhibit the voltage-gated sodium (Na) channels in nerve membranes, thereby impeding the passage of pain signals to the central nervous system in the peripheral tissues [17]. The local anaesthetic agents that are common are lidocaine, articaine, mepivacaine, prilocaine and bupivacaine, which are described by various times of onset, strength and time of action [8]. Vasoconstrictors such as epinephrine help to prolong the anaesthetic effect by adding to the effect because there is a minimal entry of the drug into the system through the injection site [18]. The selection and dosing of these agents are important in giving good analgesia with minimal adverse reactions or systemic toxicity [19]. The awareness of the pharmacological properties, including the metabolism and elimination pathways, enables clinicians to modify the administration of anaesthetics depending on the health condition and requirements of the patient [20]. Further studies on the fields of drug interaction and pharmacokinetics have enhanced the safety profile of dental anaesthetic agents currently in use [21].

In spite of the notable progress of dental anaesthetic methods, various challenges have been encountered in the attainment of optimal pain management and patient safety in the course of carrying out a dental procedure [22]. The difference in health conditions of patients and psychological aspects, such as dental anxiety and pain perception, are some of the factors that could alter the perception of the delivery of anaesthesia [23]. The success rate of the anaesthetic techniques in clinical practice could also be influenced by procedural complexity, the experience of operators and anatomical variation [24]. Besides this, it is vital to have the safety of patients as a priority, and this is achieved by observing them keenly, dosage selection and adherence to the guidelines that have been given during the treatment process. Continuous studies and the development of superior clinical practices are hence central in enhancing decision-making in dental anaesthesia. This review is an attempt to synthesise the current knowledge about patient factors, procedural considerations and outcome measures to aid in more effective and evidence-based anaesthetic choice in the dental practice.

Objective of the review

The objective of this review is therefore to discuss the clinical decision-making processes that are involved in dental anaesthesia by combining patient-related factors, complexity in the procedure and outcome measures for anaesthesia. It aims to summarise the current evidence relating to anaesthetic techniques and pharmacological agents and safety practices used in dentistry and highlight the issues that support the provision of good pain control, enhanced safety and optimised treatment outcomes during a dental procedure.

Methodology

This narrative review was conducted to summarise current evidence on clinical decision-making in dental anaesthesia. A literature search was performed using PubMed, Scopus, Web of Science and Google Scholar for studies published between 2015 and 2025. Search terms included 'dental anaesthesia', 'local anaesthesia', 'dental pain management', 'sedation in dentistry', 'anaesthetic agents', 'patient assessment' and 'anaesthetic complications'. Peer-reviewed clinical studies, reviews, case-based reports and guidelines relevant to anaesthetic techniques, pharmacological agents, patient factors, procedural complexity, monitoring, safety and outcomes were included. Non-English articles, conference abstracts, editorials and studies unrelated to dental anaesthesia were excluded. No systematic literature review, meta-analysis or statistical analysis was performed. Findings were narratively synthesised to provide an integrated clinical overview.

## Review

Biological basis of pain and anaesthetic dentistry

Dental pain arises from the specialised nociceptors in the dental pulp, dentin, periodontal ligament and surrounding oral tissues that respond to thermal, mechanical and chemical stimuli by triggering neural signalling to the central nervous system [16]. Sensory impulses produced in these receptors are carried by afferent nerve fibres that converge within the trigeminal nerve, which is considered the primary neural conveying system that is involved in the conduction of pain impulses from the oral cavity to the brainstem and higher centres of the cerebral cortex [17]. On the activation of nociceptors during inflammatory conditions of the dentition, such as pulpitis, chemical mediators such as prostaglandins and bradykinin are released, which amplify nerve excitability and pain perception [5]. This inflammatory sensitisation has an important lowering effect on the activation threshold of nociceptive fibres and is a part of the intense dental pain that is experienced during infection or tissue injury [10]. Peripheral sensitisation resulting from the action of inflammatory mediators further enhances the responsiveness of the nerves and makes it probable that stimuli that are not normally painful will be experienced as a painful sensation [20].

Dental pain is transmitted by the trigeminal nerve along complex pathways that relay the sensory information from the oral tissues in the trigeminal ganglion to the brainstem sensory nuclei [21]. Signals going through these pathways are processed and integrated in the central nervous system before they reach cortical areas responsible for the perception of conscious pain [2]. The repeated or sustained stimulation of nociceptive fibres may lead to central sensitisation in the trigeminal nucleus, which causes an exaggerated reaction to dental stimuli [13]. Central sensitisation is responsible for long-lasting pain sensations and can be a reason for the ineffectiveness of traditional anaesthetic methods applied to inflamed dental tissues [24]. These neural processes have shown how injury to the peripheral tissue and the central nervous system interact to affect the intensity and duration of dental pain [25].

**Figure 1:**
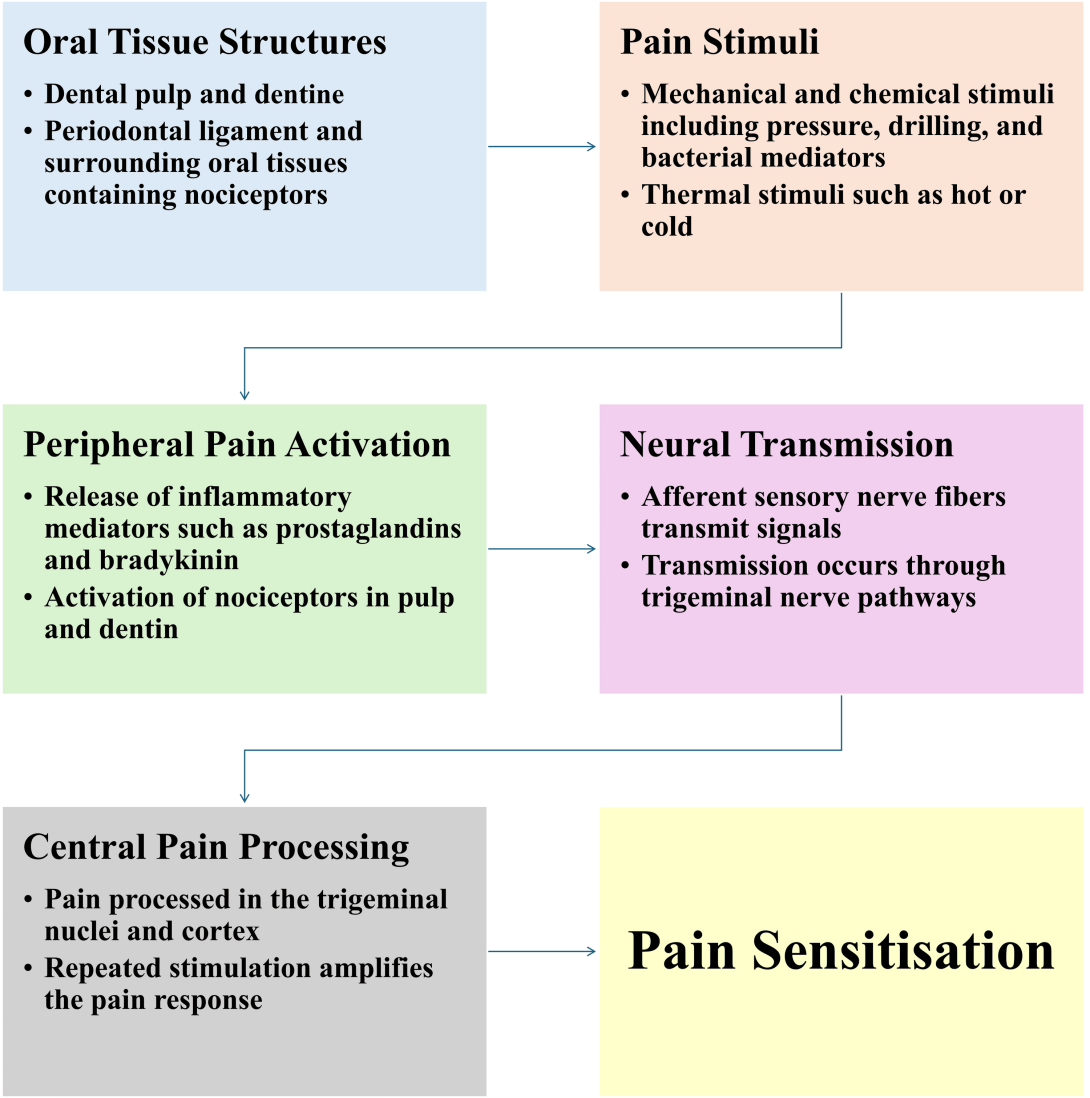
Mechanism of dental pain generation and neural processing

Understanding the neurophysiological processes involved in dental pain is necessary to choose the method of anaesthesia that breaks the conduction of nerves during dental procedures [26]. Local anaesthetic agents operate on the principle of blocking the voltage-gated sodium channels in neuronal membranes, which stops the conduction of action potentials along sensory nerves [27]. This pharmacological break in the information transmission in the nervous system causes a reversible loss of feeling in the affected area and makes it possible to execute dental treatments without causing pain [28]. Tissue pH, the diameter of nerve fibre and the extent of myelination are factors affecting the efficacy of nerve block given by local anaesthetic agents [29]. The efficient knowledge of these biological mechanisms allows clinicians to optimise the anaesthetic strategy and provide better comfort to the patient during dental treatment [30]. Figure 1 shows the mechanism of pain generation in the teeth through neural pathways in the trigeminal nerve.

Classification of dental anaesthesia techniques

Dental anaesthesia techniques are categorised according to the degree of analgesia obtained and the procedures used to deliver anaesthetic agents during dental procedures [31]. Local anaesthesia is the most common method used in routine dental practice as it gives focused pain control without the loss of awareness or protective reflexes [32]. Infiltration anaesthesia is when the anaesthetic solution is injected directly into the tissues around the tooth and prevents transmissions of pain signals from terminal nerve endings [33]. Nerve block techniques involve the deposition of the anaesthetic solution close to a major nerve trunk, such as the inferior alveolar nerve, to cause anaesthesia in a much larger anatomical area [34]. These techniques enable clinicians to provide effective analgesia during a large array of dental procedures, including restorative treatment and extractions [35].

Supplementary injection techniques are frequently used in cases of inadequate anaesthesia with either conventional infiltration or nerve block techniques [36]. In periodontal ligament injections, the anaesthetic solution is injected directly into the periodontal space around the tooth [37]. Intraosseous anaesthesia is a technique whereby the anaesthetic solution is injected into the cancellous bone next to the tooth, in which it diffuses into the surrounding tissues in a short period of time [38]. Interseptal injections are done into the interdental bone and also provide localised anaesthesia for periodontal and restorative procedures [39]. These supplementary methods are used to increase the effectiveness of the anaesthetic method in these situations where inflammation or anatomical variation restricts conventional techniques [40].

Sedation techniques form another significant category of dental anaesthesia to reduce the patient's anxiety and enhance the tolerance of the procedure [41]. Nitrous oxide-oxygen inhalation sedation is widely used as it produces mild sedation with rapid onset and recovery [42]. Oral conscious sedation and intravenous sedation are used in patients who need deeper levels of relaxation for complex dental procedures [43]. General anaesthesia is normally reserved for hospital-based settings where large amounts of oral surgery or treatment of uncooperative patients are necessary [44]. The selection of the correct anaesthetic method is conditioned by patient health status, the complexity of the procedure and the probable length of treatment [16].

Pharmacological agents used in dental anaesthesia

Local anaesthetic agents used in dentistry belong mostly to the amide class due to their better chemical stability and a reduced incidence of hypersensitivity reactions in comparison to ester-based anaesthetics [41]. Lidocaine is currently one of the most used drugs in dental surgery for routine dental procedures because of its fast onset, intermediate duration of effect and good safety in clinical practice [18]. Articaine has received growing interest due to its greater lipid solubility and better diffusion ability through bone tissues that enable the successful use of infiltration anaesthesia, especially in maxillary operations [33]. Other amide anaesthetics such as mepivacaine and prilocaine are also frequently used based on clinical needs and the length of the time of analgesia [20]. Bupivacaine is usually chosen in situations when longer periods of postoperative pain relief are needed since it causes a longer duration of sensory blockade than other local anaesthetic agents [27].

The pharmacological action of local anaesthetic agents is a reversible blockade of voltage-gated Na channels contained within the nerve cell membranes, which prevents the initiation and spread of the excitation along sensory nerve cells [22]. By inhibiting the influx of sodium on neuronal depolarisation, these agents effectively interrupt nociceptive transmission from the peripheral tissues to the central nervous system [31]. The efficiency of this mechanism is dependent on many factors such as lipid solubility, protein-binding capacity and the pKa of the anaesthetic agent [37]. Highly lipid-soluble drugs show a higher level of penetration into the nerve membranes, and more potent anaesthetic effects are generally reported [24]. Further, the extent of protein binding affects the duration of anaesthetic action by affecting the duration of drug binding to the neural tissues [40].

Vasoconstrictors are often added to dental anaesthetic formulations to increase their clinical value and the duration of their analgesic action [16]. Epinephrine is the most commonly used vasoconstrictor as it causes decreased blood flow at the injection site and decreased systemic absorption of the anaesthetic agent [34]. Reduced vascular absorption allows higher local concentrations of the anaesthetic drug and therefore an increased depth and duration of anaesthesia during dental procedures [29]. Vasoconstrictors also play a role in the enhanced hemostasis by limiting bleeding within the operative field, which increases visibility during surgical interventions [42]. Adjunct pharmacological agents such as analgesics, anti-inflammatory medications and sedatives may also be injected with local anaesthetics to enhance pain control and patient comfort during dental treatment [21]. Table 1 summarises the commonly used local anaesthetic agents in dentistry, including their concentration, duration of anaesthetic action and major clinical use.

**Table 1:** Common local anaesthetic agents used in dentistry

Local Anaesthetic Agent/Class	Typical Concentration	Duration of Anaesthesia	Main Clinical Use	Reference
Local anaesthetic agents (amide group)	Variable	Procedure-dependent	General dental pain control	[17]
Lidocaine	2% with epinephrine	60-120 minutes	Routine dental procedures and extractions	[16]
Articaine	4% with epinephrine	60-180 minutes	Infiltration anaesthesia, especially in maxillary procedures	[33]
Mepivacaine	2%-3%	60-120 minutes	Procedures where vasoconstrictors are contraindicated	[20]
Bupivacaine	0.5% with epinephrine	180-480 minutes	Long-duration procedures and postoperative analgesia	[27]

Patient factors affecting anaesthetic selection

Patient-related characteristics are a basic component of how anaesthesia technique and drug selection are determined within dental practice [36]. Age is one of the key determinants since there are physiological differences between paediatric and geriatric populations, which affect drug pharmacokinetics and pharmacodynamics [17]. Children often need lower dosages of anaesthetics and behavioural management strategies to ensure their safe and effective treatment [43]. In contrast, the hepatic and renal function in elderly patients might be diminished, leading to changes in the metabolism and clearance of anaesthetic agents employed during the dental procedure [19]. These physiological differences in turn require adequate dose adjustments and monitoring throughout treatment [30].

Systemic health conditions also need to be considered during the choice of dental anaesthetic agents [38]. Patients with cardiovascular disorders may need to be limited in the use of vasoconstrictors due to their effect on heart rate and blood pressure [22]. Diabetes mellitus and other metabolic disorders may affect the tissue healing process and predispose to postoperative complications after dental procedures [41]. Respiratory disorders can be a complication of sedation procedures, and therefore, a careful evaluation of the airway and oxygenation status is necessary before treatment [25]. A thorough assessment of medical history may help clinicians to avoid drug interactions and identify contraindications associated with certain anaesthetic agents [34].

Psychological and behavioural variables play an important role in the patient's response to dental anaesthesia and pain perception [18]. Dental anxiety and fear can increase perceived pain intensity and decrease the cooperation of patients during treatment procedures [44]. Sedation methods, as well as behavioural management techniques, are frequently implemented to manage patient anxiety and enhance their tolerance to dental interventions [28]. Individual differences in pain threshold and psychological stress response may also have an impact on the success of anaesthetic methods [33]. The comprehensive evaluation of the patient enables clinicians to individualise anaesthetic strategies based on the needs of each individual patient and help improve clinical outcomes [20]. Table 2 highlights patient-related factors influencing anaesthetic choice in the dental practice, as well as important clinical considerations.

**Table 2:** Patient factors influencing anaesthetic selection

Patient Factor	Clinical Consideration	Impact on Anaesthetic Choice	Management Strategy	Reference
Paediatric age	Behavioural response and sedation requirement	Selection between sedation or general anaesthesia	Clinical assessment using behavioural scales	[3]
Cardiovascular conditions	Sensitivity to epinephrine in anaesthetic solutions	Careful vasoconstrictor use	Monitoring cardiovascular response	[1]
Medical conditions	Presence of systemic diseases affecting dental care	Adjustment of treatment planning	Case-based clinical management	[2]
Diabetes mellitus	Changes in blood glucose during dental extraction	Monitoring metabolic status	Controlled anaesthetic administration	[29]
Dental anxiety	Fear and anxiety related to dental treatment	Need for behavioural or psychological support	Anxiety management techniques	[26]

Procedural complexity and anaesthetic planning

The complexity of dental procedures is a major determinant in the most appropriate anaesthetic technique and pharmacological approach [37]. Preventive and diagnostic dental procedures usually need minimal anaesthesia because of limited tissue trauma during these types of interventions [21]. Restorative procedures, including cavity preparation for most cases, require the use of infiltration anaesthesia to block sensory nerve endings that surround the affected tooth [16]. Endodontic treatment frequently requires the profound anaesthesia of the pulp because of the severe inflammatory pain of irreversible pulpitis [40]. Good anaesthetic planning will ensure that there are adequate analgesia and no discomfort from anaesthesia [31].

In surgical dental procedures where there is much tissue handling, a greater depth and extent of anaesthetic effect is needed [27]. Periodontal surgeries and the placement of dental implants often require regional nerve block techniques in order to be able to anaesthetise a larger anatomical area [23]. Oral and maxillofacial surgeries such as third molar extractions usually require a very deep nerve block for complete pain relief within the surgical field [42]. The expected duration of the procedure is also considered when choosing anaesthetics since longer procedures require anaesthetics with a longer duration of action [34]. The selection of appropriate anaesthetic agents allows for analgesia to continue throughout the procedure without having to be repeatedly injected [19].

Anatomical considerations are also important in anaesthetic planning in dental procedures [24]. The thickness of the cortical bone in the posterior mandible often reduces the efficacy of infiltration anaesthesia and hence requires inferior alveolar nerve block techniques [38]. Anatomical differences in the location of nerves may influence the success rates of the traditional injection methods [30]. Supplemental anaesthetic approaches such as intraosseous or periodontal ligament injections can be needed when regular approaches are unsuccessful in providing adequate analgesia [41]. The careful assessment of the complexity of the procedure and the anatomy enables anaesthesia to be optimised and provide comfort to the patient [22]. Table 3 presents the relationship between the complexity of the dental procedure and the recommended anaesthetic techniques adopted in clinical practice.

**Table 3:** Anaesthetic techniques according to procedural complexity

Dental Procedure	Anaesthetic Technique	Duration Requirement	Reference
Preventive and routine dental treatment	Local anaesthesia or minimal anaesthesia	Short	[19]
Restorative dental procedures	Local infiltration anaesthesia	Moderate	[20]
Endodontic treatment with pulpitis	Inferior alveolar nerve block	Moderate	[21]
Dental implant surgery	Regional nerve block techniques	Long	[11]
Third molar extraction	Local anaesthesia with postoperative pain management	Long	[15]
Advanced surgical procedures	Regional anaesthesia approaches	Long	[24]

Risk assessment and pre-anaesthetic evaluation

Risk assessment is an essential part of dental anaesthesia because it allows treatment complications to be recognised before the treatment commences [18]. A detailed review of the patient's medical history gives information on systemic diseases, allergies, medications and past reactions to anaesthetic agents [36]. The identification of such factors assists clinicians in adjusting treatment strategies to reduce the risk of adverse reactions [29]. The comprehensive evaluation of the patient therefore plays a critical role in ensuring the safe administration of anaesthesia during dental procedures [24].

The American Society of Anesthesiologists (ASA) physical status classification system is widely used for the assessment of patients' health status before the administration of anaesthesia [39]. Patients under a higher ASA class may need extra care or treatment under hospital-based facilities and not routine dental clinics [20]. The evaluation of airway anatomy and respiratory status is especially important in any sedation or general anaesthesia [34]. The assessment of cardiovascular function is also useful in helping clinicians determine if solutions containing vasoconstrictors can safely be given with anaesthetic solutions [42]. These evaluations help clinicians anticipate the possibility of complications and carry out preventive strategies [27].

Medical consultation with physicians might be required when managing patients with complicated systemic conditions [16]. Diagnostic investigations such as laboratory testing may be needed to determine whether a patient has any bleeding problems, metabolic diseases or organ dysfunction that may affect the safety of the anaesthetic [41]. Careful preoperative planning is an aid to clinicians in choosing to deliver the safest anaesthetic agents and techniques for the individual patient [21]. Thorough risk assessment reduces the occurrence of complications and improves the outcome for the patients during dental treatment [33].

Intraoperative monitoring and safety techniques

The continuous observation of physiological parameters during dental procedures adds to patient safety when anaesthesia or sedation is given [40]. Vital signs such as heart rate, blood pressure, respiratory rate and oxygen saturation provide important indicators of patient stability throughout treatment [23]. The monitoring of these parameters allows clinicians to detect early signs of adverse reactions or complications of the use of anaesthetic drugs [34]. The early detection of physiological changes aids in the intervention and prevention of major medical emergencies in a short period of time [18].

Modern dental clinics have special monitoring equipment to monitor the physiological status of the patients during procedures [29]. Pulse oximetry is a very common technique used to monitor the saturation of oxygen and evaluate the respiratory function when sedation has been administered [31]. Automated blood pressure monitors allow the clinician to examine the cardiovascular stability during a long dental procedure [41]. Capnography may also be used in some clinical situations in order to monitor ventilation and detect respiratory depression during sedation [22]. These technologies make dental anaesthetic practices much safer to a great extent [37].

**Figure 2:**
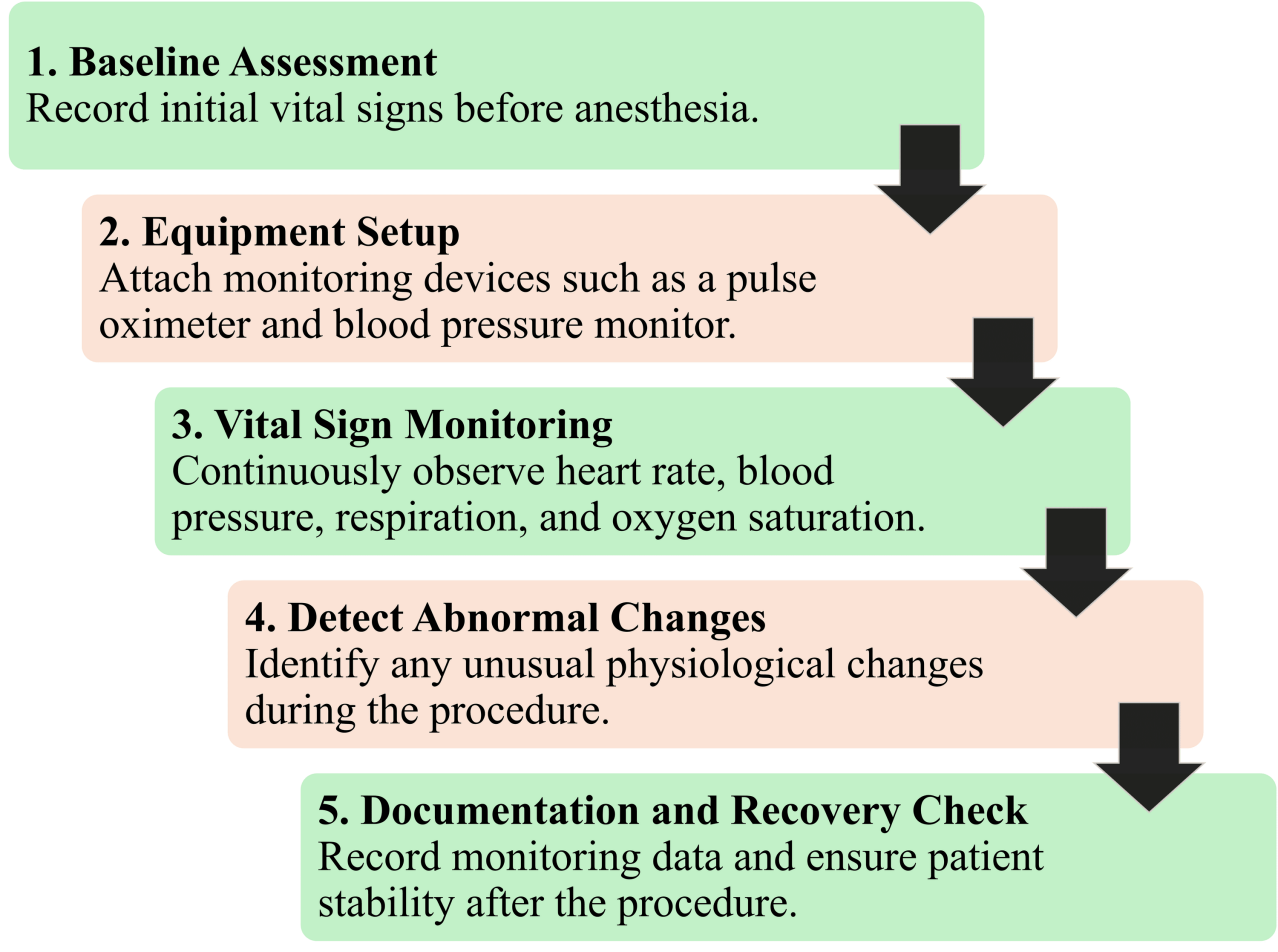
Five-step monitoring process during dental anaesthesia

Another important intraoperative safety topic in dentistry is emergency preparedness [24]. Dental clinics need to have easily accessible emergency equipment such as oxygen delivery systems, airway management equipment and resuscitation devices [42]. Staff training in emergency management procedures allows for a quick response in the event of anaesthetic complications [30]. Clinical record-keeping and medicolegal protection also require the accurate documentation of anaesthetic administration and monitoring [19]. In the implementation of structured monitoring protocols, the whole dental anaesthetic procedure can be much safer [36]. The five-step monitoring process that is followed during dental anaesthesia to ensure the safety of patients is presented in Figure 2.

Complications associated with dental anaesthesia

The use of dental anaesthesia is normally safe; complications may sometimes occur during or after anaesthetic administration [25]. Local complications include pain at the injection site, hematoma formation and transient nerve injury due to improper injection technique and accidental vascular puncture [38]. Hematomas are formed when blood vessels are damaged during injection, resulting in the localised bleeding and swelling of surrounding tissues [33]. Temporary nerve damage can have symptoms such as numbness, paraesthesia or changes in the sensation of oral tissues [21]. Careful injection technique and the knowledge of anatomy minimise the risk of these local complications [29]. Systemic complications may occur when excessive amounts of anaesthetic agents enter the systemic circulation [16]. Local anaesthetic systemic toxicity may be first manifested by neurological symptoms such as dizziness, tinnitus, confusion or seizures [41]. Severe cases may develop into cardiovascular complications such as arrhythmias, hypotension or cardiac arrest [22]. The timely identification and treatment of systemic toxicity are critical to avoid life-threatening consequences [34]. Strict adherence to recommended dosages helps minimise the risk of systemic toxicity [20].

Allergic reactions to the dental anaesthetic agents or to preservatives contained in anaesthetic formulations are rare but possible [27]. Hypersensitivity reactions can be skin rashes, swelling, respiratory distress or, in severe cases, anaphylaxis [42]. Immediate management by appropriate emergency medications is required in cases of allergic reactions to dental procedures [18]. Thorough patient history evaluation and allergy screening help practitioners to avoid exposure to potentially harmful agents [36]. Proper preparation and adherence to clinical protocols go a long way in reducing the risk of anaesthetic complications [31].

Outcome measures: dental anaesthesia

The evaluation of outcomes of dental anaesthesia is primarily directed to the success of the pain control achieved during dental procedures [43]. Successful anaesthesia enables clinicians to carry out procedures without any discomfort or distress to the patient [20]. Objective indicators such as the lack of pain response during operative treatment are evidence of adequate anaesthetic effectiveness [31]. Patient-reported pain scores are also commonly used in an attempt to measure the subjective experience of analgesia in dental care [38]. These measurements assist clinicians in assessing the clinical performance of various anaesthetic techniques [24]. Patient satisfaction is an important outcome measure because positive treatment experiences enhance compliance with dental care over the long term [16]. Comfortable dental procedures decrease patient anxiety and increase the desire to return for future dental procedures once needed [42]. Good communication between dentists and patients also helps in providing better treatment satisfaction and perceived quality of care [29]. Minimising intraoperative pain and postoperative pain improves the experience of dental treatment [35]. These factors jointly determine the patient perception of the effectiveness of dental anaesthesia [18].

Additional outcome measures include the success rate of the procedure, complications after the procedure and recovery time after dental treatment [22]. The rapid return of normal oral function following anaesthesia is a positive indication of appropriate drug selection and dosing during the treatment [34]. The monitoring of the outcome of surgery is important to assist clinicians in identifying potential improvements in anaesthetic techniques and patient management strategies [27]. The continuous assessment of clinical outcomes aids evidence-based practice and enhances the safety of dental anaesthetic procedures [41]. Outcome assessment, therefore, has a critical role in furthering the practices of modern dental anaesthesia [23].

Limitations and future directions

There are a few limitations to this review. The synthesis is based on previously published studies, which differ in methodology, sample size and clinical settings. Differences in anaesthetic techniques, in patient populations and in the reporting of outcomes may have an impact on the comparability. The limited availability of long-term clinical data on dental anaesthetic strategies also limits a full evaluation of sustained safety and effectiveness data.

Future research should be oriented towards standardised outcome measures to improve comparability across studies. Large multicentre clinical trials are required to evaluate anaesthetic agents and techniques across different patient populations and dental procedures. Further investigation into personalised anaesthetic approaches, including patient-specific risk assessment and pharmacological response, might help improve treatment planning. The integration of digital tools and clinical decision support systems has the potential to improve evidence-based anaesthetic selection and patient safety in dental practice.

## Conclusions

This review concludes that good dental anaesthesia is vital to achieving safe, comfortable and successful dental procedures. Dental pain occurs due to complex neurophysiological mechanisms, and these involve the activation of nociceptors of oral tissues, the transmittance of the signals through pathways of the trigeminal nerves and processing within the structures of the central nervous system. Understanding these biological mechanisms provides a scientific foundation for selecting anaesthetic strategies in dental practice. Various anaesthetic techniques, such as local anaesthesia, sedation and general anaesthesia, are important depending upon patient characteristics and procedural requirements. Local anaesthetic agents include lidocaine, articaine, mepivacaine and bupivacaine, all of which are still used widely because of their reliability in blocking nerve conduction and preventing pain perception in the course of treatment in dentistry. The selection of anaesthetic methods should take into account patient-related factors such as age, systemic health and psychological conditions and procedural factors such as the complexity of treatment and expected duration. Patient safety during dental anaesthesia is supplemented by strict requirements in pre-anaesthetic assessment, as well as the continuous intraoperative monitoring of physiological parameters. The monitoring of vital signs and the detection of the initial signs of abnormalities minimise the risk of complications and enable early clinical intervention. The integration of evidence-based clinical decision-making with progress in monitoring tools and pharmacological development is expected to further enhance the safety and efficiency of procedures, as well as outcomes of anaesthetic practice in dentistry.
